# Bilateral femoral posterior neurocutaneous perforater flap successfully treating Fournier gangrene: A case report

**DOI:** 10.1097/MD.0000000000008720

**Published:** 2017-11-17

**Authors:** Tao Wang, Gang Zhao, Yong-Jun Rui, Jing-Yi Mi

**Affiliations:** Department of Hand Surgery, Wuxi NO.9 People's Hospital Affiliated to Soochow University, Wuxi, China.

**Keywords:** Fournier gangrene, island flap, necrotizing fasciitis, treatment

## Abstract

**Rationale:**

Necrotizing fasciitis (NF), characterized by widespread fascial necrosis, is a rare disease in clinic. Fournier gangrene (FG) is a special type of NF involved of perineum and scrotum. To our knowledge, no article has reported on bilateral femoral posterior neurocutaneous perforater flap treating for FG.

**Patient concerns:**

A 61-year-old Chinese male complained of perineal skin necrosis for 19 days. The patient received treatment in other hospital due to chronic bronchitis on April 15th and body temperature ranged from 38 to 39 °C. Then he received antiinfection therapy. Perianal cutaneous occurred mild necrosis on May 08th. And the necrosis generally deteriorated. He came to our hospital for treating necrosis in area of perineum and scrotum on May 28th.

**Diagnoses:**

He was diagnosed with FG and chronic bronchitis.

**Interventions:**

The patient underwent debridement on June 2nd and received bilateral femoral posterior neurocutaneous perforater flap on June 29th. Besides, the patient was treated with whole-body nutrition support and antibiotic treatment.

**Outcomes:**

One week after the 2nd operation, the flap showed normal color. The result shows good outcome and no recurrence of the clinical symptoms occur till now.

**Lessons:**

FG is rare. Bilateral femoral posterior neurocutaneous perforater flap is an effective procedure to treat FG. The outcome of combined therapy is satisfactory.

## 1 Introduction

In 1871, Joseph Jones was the first to report necrotizing fasciitis (NF) which was featured by rapidly progressive and widespread infection in fascia and subcutaneous tissue.^[1]^ NF is rare and leads to extensive necrosis and sepsis in serious condition,^[2]^ bringing a huge burden for patients in the field of physiology and psychology. Young and Aronoff^[3]^ reviewed literature on NF and found that rate of NF in extremities was higher than that in central areas. Previous studies^[4,5]^ reported that the risk factors for NF were diabetes, trauma, wound infections, decubitus ulcers, alcoholism, carcinoma, peripheral vascular disease, smoking, and intravenous drug abuse. NF could be infected by various kind of bacteria.^[6]^ In 1883, Alfred Jean Fournier firstly put forward concept of Fournier gangrene (FG), as a specific type of NF, that NF occurred in perineum and scrotum.^[7]^ Recent articles indicated that mortality rates for NF varied from 6% to 76%.^[8]^ However, the mortality rate of FG has been reported as low as 16%.^[9]^ Perineum and scrotum could be early observed and early treatment explaining the reason for this low mortality rate of FG. Early debridement is beneficial for the patients with NF. Several authors^[10,11]^ reported using the pudendal thigh flap as an axial-pattern flap based to repair wound. El-Khatib^[12]^ used V-Y flap to repair FG. We report a new surgical procedure, bilateral femoral posterior neurocutaneous perforater flap, to treat FG.

## 2 Consent

The current study was approved by the patient for publication of this case report and any accompanying images and ethics committee of the Wuxi NO.9 People's Hospital Affiliated to Soochow University.

## 3 Case report

**Figure 1:**
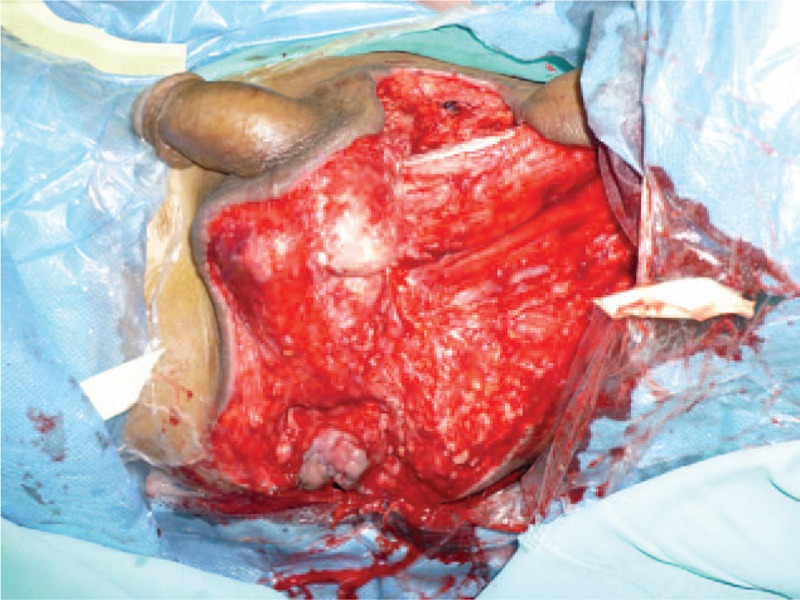
Wound in perineum and scrotum area.

**Figure 2:**
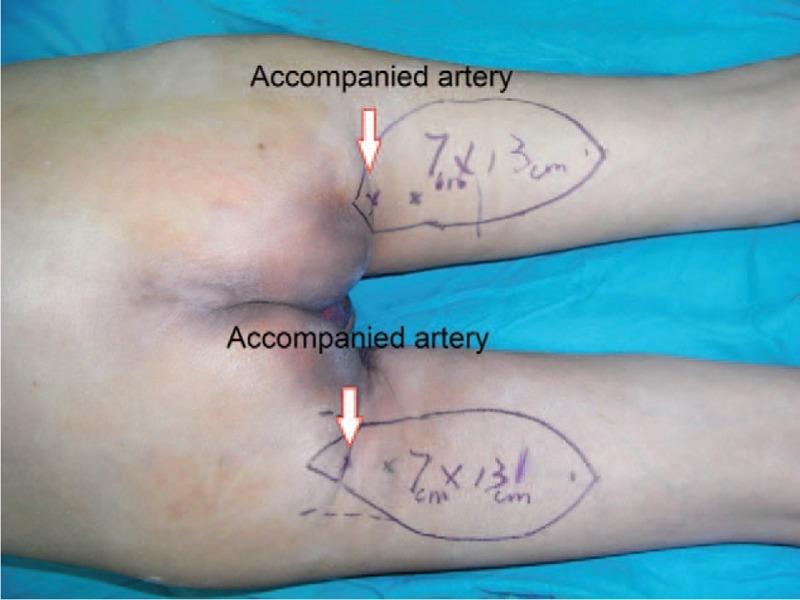
We designed a 7 cm × 13 cm of flap.

**Figure 3:**
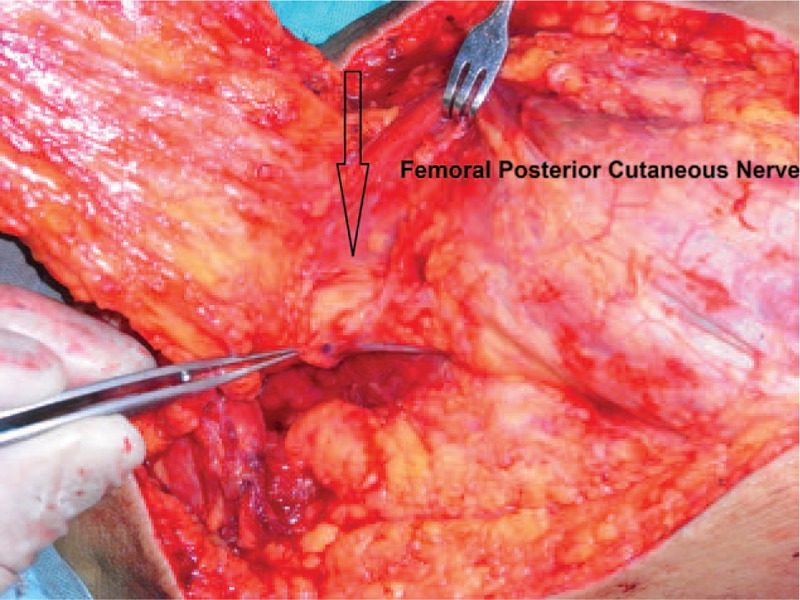
The femoral cutaneous nerve in this flap.

**Figure 4:**
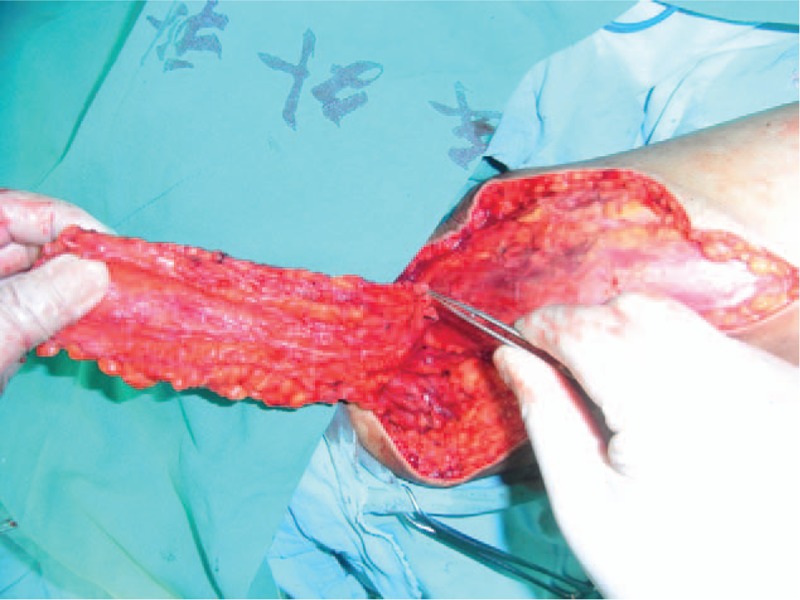
Cut femoral cutaneous nerve and accompanied artery and ligate accompanied artery.

**Figure 5:**
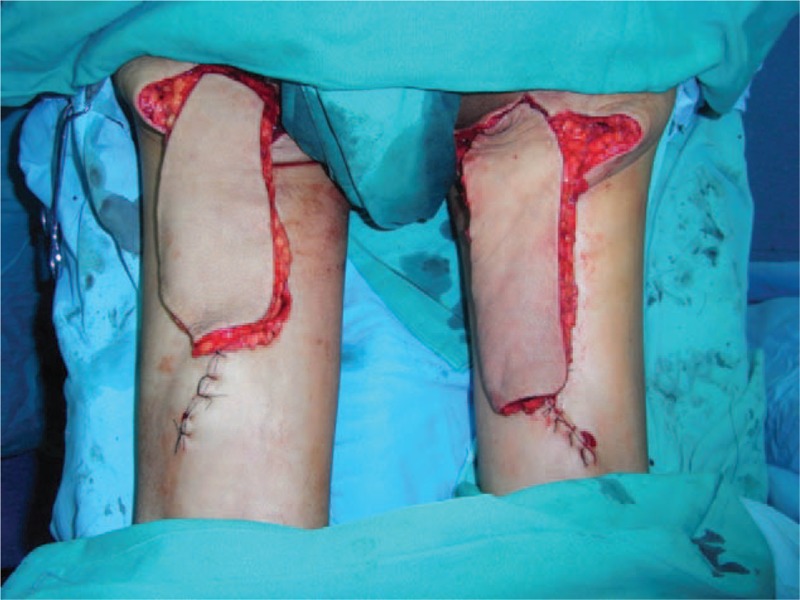
Lift the flap, expose lower margin of gluteus maximus and retain 5 cm width of deep fascia.

**Figure 6:**
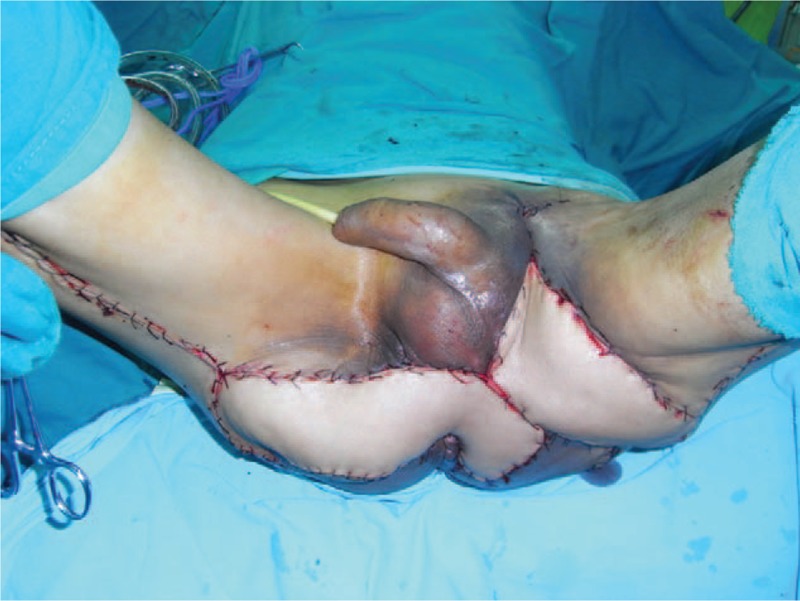
Right flap was transferred to cover perineal region and left side was transferred to cover scrotum.

A 61-year-old adult man complained about perineal skin necrosis for 19 days. The patient received treatment in other hospital due to chronic bronchitis on April 15th and body temperature maintained from 38 to 39 °C. Then he received antiinfection therapy. Perianal cutaneous occurred mild necrosis on May 08th. Patient came to our hospital for treating perineal skin necrosis on May 28th. The hemogram on May 29th showed white blood cell: 9.3 × 10^9^/L, red blood cell (RBC): 3.4310^12^/L, total protein (TP): 43.5 g/L, and albumin (ALB): 19.7 g/L. Lung X-ray presented bronchitis changes. Bacteria culture of wound exudates at the first time indicated that wound was infected by *Klebsiella pneumoniae* which was sensitive to piperazilin. From Fig. 1, we could clearly see huge wound in the area of perineum and scrotum. That patient was diagnosed with FG and chronic bronchitis. We performed adequate debridement for this patient and used vacuum sealing drainage (VSD) for healing wound on June 2nd and the patient was treated with piperazilin and whole-body nutrition support after surgery. The hemogram on June 25th showed white blood cell: 6.9 × 10^9^/L, RBC: 3.9 × 10^12^/L, TP: 55.3 g/L, ALB: 26.8 g/L, and body temperature maintained from 36 to 37 °C. After observing the wound for nearly a month, we found that wound was in good condition and we decided to operate bilateral femoral posterior neurocutaneous perforater flap on June 29th. We designed a 7cm × 13 cm of flap and regarded midpoint of bilateral natal cleft as rotation point and posterior thigh midline as axis, shown in Fig. 2. The diameter of artery accompanied cutaneous nerve was 1.2 mm by using Doppler before surgery. We cut into the deep fascia and exposed femoral cutaneous nerve (Fig. 3), then ligated the femoral cutaneous nerve and accompanied artery at distal end (Fig. 4). Lifing the flap from distal to proximal end, we exposed lower margin of gluteus maximus and retained 5 cm width of deep fascia including femoral cutaneous nerve and accompanied artery (Fig. 5). At last, the right flap was transferred to cover perineal region and the left to cover scrotum (Fig. 6). Bacteria culture of wound exudates during operation indicated that wound was infected by *Klebsiella oxytoca* which was sensitive to piperazilin. The patient was treated with piperazilin and whole-body nutrition support after surgery.

## 4 Discussion

Our case presented a 61-year-old man with skin necrosis in section of sperineum and scrotum for 19 days, as shown in Fig. 1. X-ray of lung showed bronchitis changes and body temperature ranged from 38 to 39°C. The patient was in poor condition due to low RBC, TP, and ALB. Bacteria culture implied wound was infected by *Klebsiella pneumoniae*. We performed adequate debridement, antibiotic therapy, and whole-body nutrition support after surgery. Observing wound in good condition, we performed bilateral femoral posterior neurocutaneous perforater flap and the outcome was satisfactory. FC is a rare disease in clinic and it is tough to cure. Recent researches^[5]^ have shown that aggressive surgical treatment including wide debridement and VSD should be considered as soon as possible to struggle for a better chance for reconstructive surgery. Mizuguchi et al^[13]^ reported a case on negative-pressure wound therapy successfully treating NF after rectal surgery. A 58-year-old man developed NF in the area of pelvis and thigh for 3 years after rectal surgery. Extensive debridement and application of negative-pressure wound therapy successfully contributed to wound bed cleansing. Baek et al^[14]^ considered differences between male and female genital anatomy, using internal pudendal artery perforator flap to treat a female patient with FG. Taking huge wound in perineum and scrotum into account in our case, we designed bilateral femoral posterior neurocutaneous perforater flap to reconstruct wound. As far as we know, no report on this surgical procedure treating FG. There were following merits. First, compared to unilateral flap, double flaps needed less smaller area to cover surface of wound and skin tension of the supplied area was smaller which was easy to suture skin of wound. Second, from Fig. 1, we could see huge wound in perineum and scrotum. The right flap was transferred to cover perineal region and the left one was to cover scrotum, which was easy to cover wound surface. However, unilateral flap was difficult to achieve the same result. The last but not the least, bilateral femoral posterior neurocutaneous perforater flap not only supplied nerve nourishment, but also provided blood supply and feeling for the recipient area. Additionally, it could offer double nerve and blood vessel which was easy to survive. Although this procedure provided several advantages, it had some limitation. First, it needs a long follow-up to prove efficacy; 2nd, we need more cases to assess this procedure.

In conclusion, FC is rare in clinic. No report has reported bilateral femoral posterior neurocutaneous perforater flap treating FG. The new surgical procedure is an effective treatment and has advantages in field of wound repair, neurotrophy, and blood supply. We provide a method for surgeons when facing the rare case like this, and we need further study to observe efficacy in long-term follow-up.
